# COVID-19 and food security in Africa: Building more resilient food systems

**DOI:** 10.12688/aasopenres.13078.1

**Published:** 2020-06-26

**Authors:** Helena Shilomboleni

**Affiliations:** 1 CGIAR Research Program on Climate Change, Agriculture and Food Security (CCAFS) East Africa, International Livestock Research Institute, Nairobi, P.O.Box 30709, Kenya

**Keywords:** Food security, COVID-19, food systems resilience, Africa

## Abstract

The COVID-19 pandemic has exposed the fragility of our food systems. Despite increased efficiencies in producing and supplying large volumes of food, our current food systems have generated multiple adverse outcomes comprising high greenhouse gas emissions, persistent hunger, and livelihood stress for farmers around the world. Nowhere else than in Africa have large numbers of people experienced more acutely these adverse shocks emanating from our food systems. Thus, building more resilient African food systems, which take a radical change of direction, is fundamentally a matter of survival. While there is broad consensus around a need for transformational change in food systems, what that entails is not always clear, and there are divergent views amongst experts on how to re-orient research priorities and agricultural solutions in ways that effectively address hunger and inequality while also protecting agrobiodiversity and the environment more broadly. This article engages with this debate and proposes an agricultural research for development agenda in Africa that balances technology transfer with realigning societal values, institutional arrangements, and policy decision-making towards the realization of greater sustainability and inclusive outcomes.

## Introduction

Various actors point to threats of a looming global food crisis ^1^ due to the impacts of the novel coronavirus (COVID-19). In major African cities such as Nairobi, Kinshasa and Lagos where up to two-thirds of the population rely on the informal sector for their livelihoods, millions of people have been left without income to purchase food due to the abrupt loss of jobs that often provide daily earnings. In rural areas where agriculture is the main source of people’s livelihoods, disruptions to transportation and logistics have made it difficult for producers (farmers, livestock keepers, fisherfolk) to sell their produce and to gain access to agro-veterinary inputs and services.

In Kenya, for instance, a country-wide curfew (7 PM-5 AM) and movement ban in and out of four counties, including Nairobi, have seen smallholder farmers who produce over 70% of the food consumed in the country face high transportation fees to deliver their produce to cities ^2^ while others scramble to find alternative markets. The effects of these restrictions might result in higher food prices, akin to experiences from the Ebola crisis in West Africa in 2014, which disrupted agricultural supply chains. Today, the COVID-19 pandemic puts a further strain on Africa’s agricultural sector which is already facing unfavorable climate change patterns involving a higher frequency and intensity of extreme weather events such as droughts and floods, market and price volatility, and the recent desert locust outbreak in the Horn of Africa.

To address the immediate food security shocks brought about by the COVID-19 pandemic, multiple African governments have introduced relief measures to cushion the poorest and most vulnerable segments of their populations as well as to ensure that producers have affordable access to farm inputs.

## Food system resilience paradigms

Beyond tackling the immediate concerns surrounding health and food emergencies, global food security leaders reiterate that the COVID-19 crisis offers an opportunity for decisive collective action towards building resilient food systems that enhance ecological sustainability and equitable outcomes ^3^. The COVID-19 outbreak has brought to the fore some of the existing challenges facing our food systems. For example, while current food systems have become efficient at producing and supplying large volumes of food, they have generated multiple adverse outcomes comprising high greenhouse gas emissions, persistent hunger, and livelihood stress for farmers around the world ^4^.

Nowhere else than in Africa have large numbers of people experienced more acutely these adverse shocks emanating from our food systems. Thus, building more resilient African food systems, which take a radical change of direction, is fundamentally a matter of survival. African Ministers of Agriculture, speaking on the impact of COVID-19 on food security and nutrition in Africa, have emphasized that developing sustainable and resilient food systems in Africa can address various negative influences beyond providing adequate food, including on public health, youth employment, education, economic development and social well-being ^5^.

While there is broad consensus around a need for transformational change in food systems, what that entails is not always clear, and there are divergent views amongst experts on how to re-orient research priorities and agricultural solutions ^6^ in ways that effectively address hunger and inequality while also protecting agrobiodiversity and the environment more broadly.

For some actors, resilient food systems are productive and efficient, and operate under the principles of climate-smart agriculture and sustainable intensification. Ideal food systems are also envisioned to support the inclusive participation and economic empowerment of especially marginalized food producers and agricultural workers such as through favorable integration into food value chains ^7^. For other actors, resilient food systems promote diversified agroecological farming and landscapes, based on the principles of food sovereignty, and are intended to produce diversified outputs ranging from increased dietary diversity from locally produced food, minimal shocks from crop and market failures, and autonomy over how resources are used ^8^. Further, it brings greater scrutiny and critical debate to normative assumptions surrounding food security as well as to the political capital and priorities that lock-in place current unsatisfactory practices in food systems ^9^.

Both of these food systems resilience paradigms offer a wide diversity of proven agricultural technologies, practices and valuable insights that can enhance the resilience of Africa’s producers and food systems. However, agricultural technology uptake amongst producers has often proved challenging and many commendable innovations fail to expand their reach to large numbers of beneficiaries in a manner that is self-sustaining or long-lasting. There are multiple reasons for their limited impact at scale. Among them is that most efforts tend to narrowly focus on generating more produce or income from existing farms but pay relatively scarce attention to the different dimensions of agricultural systems constrains (mainly economic and institutional in nature), or whether innovations necessarily align with the specific constraints, interests and motivations of smallholder producers and other stakeholders ^10^.

## Building more food system resilience in Africa

Building more resilient food systems in Africa will require reconfigurations that balance technology transfer with realigning societal values, institutional arrangements, and policy decision-making towards the realization of greater sustainability and inclusive outcomes ^11^. This process will need to pay attention to and support the following elements:

- Offer low-cost or cost-effective agricultural innovations and practices that can enhance the resilience of Africa’s resource-constrained producers to hedge safely against risks in environments where they are routinely subject to multiple unpredictable shocks and outcomes (e.g., crop loss, market failure).
- Build the agency of individuals and communities to foster ownership in the management and control of such agricultural innovations, as well as to advocate for their own priorities and interests more effectively, beyond the short-term duration of typical agricultural development interventions.
- Engage with decision-makers to advocate for the implementation of strong institutional or policy mechanisms that support context-appropriate agricultural solutions and can enhance resilience in Africa’s food systems.
- Support research and learning that informs African food producers and consumers about the value of strengthening food systems resilience in a manner that provides nutritious and healthy food while delivering livelihood benefits to farmers and promoting sustainable agricultural practices.

When taken together, these elements can help to reposition agricultural interventions to enhance food systems resilience impact in Africa. Various actors and initiatives are already working towards this agenda. However, their efforts often face enormous structural constrains and struggle to gain the necessary political and financial support needed to meaningfully expand their impact at scale.

For example, Bioversity International and the CGIAR Research Program on Climate Change, Agriculture and Food Security (CCAFS) have supported the establishment and capacity-enhancement of community seed banks in Uganda, Kenya and Uganda ^12^. This work aims to lend greater to farmers’ social seed networks, commonly referred to as informal seed systems, which in most parts of Africa supply up to 80% of the seeds grown by smallholder farmers. These community seed banks serve multiple key functions. Among them is *in-situ* conservation of local plant genetic resources, and access to greater varietal diversity of seeds and planting materials acquired from national and regional gene banks and farmer-to-farmer exchanges. Despite this vital role that informal seed systems play in the production and distribution of a vast majority of the seeds used by Africa’s smallholder farmers, they are often overlooked in dominant seed system development endeavors which largely favor the expansion and commercialization of formal seed systems ^13^.

Similarly, the Alliance for Food Sovereignty in Africa (AFSA), one of Africa’s biggest civil society movements, is involved in an advocacy campaign for agroecology across Africa. Agroecology seeks to (re)design farming systems in ways that maximize agrobiodiversity using a wide range of crops, seed varieties, and farm animals, as part of a strategy to stabilize food supply against climatic variability and seasonal shortages, while building healthy agro-ecosystems ^14^. For AFSA, agroecology should focus on building upon Africa’s diverse food systems and traditional farming practices, while ensuring that farmers are in control of all aspects of food production. While AFSA has seen increased recognition in some global policy arenas, the movement faces difficulty accessing critical national and regional policy spaces that can facilitate the implementation of some of its agroecology efforts ^15^. To them, African agricultural policymaking is biased towards intensifying the production of staple crops, using a narrow range of agrochemicals and improved seeds as evident in multiple Farmer Input Subsidy Programmes ^16^.

As Africa’s policy-makers grapple with how to meet the food security demands of their nations considering disruptions caused by the COVID-19 pandemic, now is also a time to consider system-wide reconfigurations that can build greater resilience in local and national food systems. Evidence from Africa-based organizations and movements demonstrate that investing in approaches that build the agency of producers and their communities to improve their agricultural practices can guarantee the stable supply of healthy and nutritious food. These efforts can help feed Africa adequately and sustainably, but they will need much greater political and financial support.

## Data availability

No data are associated with this article.

## Footnotes

^1^ Food Security Information Network (FSIN) (2020). *2020 - Global Report on Food Crises.*

## References

[1] : Agronomy for Development: The Politics of Knowledge in Agricultural Research. *Routledge Ltd.*.2017; 10.4324/9781315284057 10.4324/9781315284057 Reference source

[2] : Adapting to Climate Uncertainty in African Agriculture.2015; 10.4324/9781315725680 10.4324/9781315725680

