# Supplementary material for: COVID-19 and food security in Africa: Building more resilient food systems
Source: AAS Open Res. 2020 Jun 26;3:27. [Version 1] doi: 10.12688/aasopenres.13078.1 (PMC7376614; doi:10.12688/aasopenres.13078.1)
Supplement: Peer review history

# Peer review: Reviewer response for version 1

**DOI:** 10.21956/aasopenres.14174.r27512

**Published:** 2020-07-22

**Authors:** Sheila Rao

**Affiliations:** 1 Carleton University, Ottawa, ON, Canada

- Overall, this article raises concerns over a looming food crisis in the Sub-Saharan Africa (SSA) region as a result of the global COVID-19 pandemic. The author argues that increasing food insecurity in both urban and rural populations is resulting from declining food production, reduction in transportation services and a loss of thousands of jobs, largely in the informal labour sector.
- The author argues for a radical shift in policy and research direction for addressing these pressing challenges and suggests building more resilient food systems that overcome underlying bottlenecks such as: improving public health services, access to education and youth unemployment. The author describes varying approaches to building resilient food systems, through food sovereignty, agro-ecology and through improved productivity and efficiency but argues that these approaches often result in narrowed outcomes that are focused on economic gains.  
- The author makes a number of recommendations for building more resilient food systems that could withstand future shocks and uncertainties, and that could mitigate the long-term impact of the current global pandemic. The article makes a strong case for shifting attention from technologically-focused innovations to cost-effective, demand driven strategies that consider individuals' and communities' agency, their control and ownership over innovations and an overall more holistic approach to multi-level decision making, learning and education.
- The author suggests that a radical or transformational change is necessary for future resilient food systems across the SSA region. The analysis could pay further attention to how substantive structural changes are needed. For example, analysis of financial and political actors that support current and future scalable and technologically-driven initiatives could identify specific investment needs around integrating resilience strategies into food systems.
- Considerations for the politics of knowledge and power relations behind agenda-setting and policy formations are also necessary to ensure that strategies are designed to meet the long-term, sustainable needs of both rural and urban dwellers in SSA. An emphasis on the processes involved in building resilient food systems could highlight approaches that include those affected the most, (for example, those based in the informal labour sector) in collaborative, co-design planning, research and implementation.
- Shocks brought on by the pandemic also pose specific risks to different actors within food value chains. Informal food vendors, material suppliers, factory workers (in food processing) for example, are all vulnerable to contracting the virus but who also risk losing their livelihoods without continuing their work in some capacity. Social protection measures are a necessary factor in establishing future food systems that benefit everyone. Further to this, context-specific, intersectional considerations (including gender, sexuality, race, class, ethnicity and able-bodyness, etc.) in the analysis could further the discussion around social and economic inequalities within food systems, and the exacerbating impact of the pandemic on the most marginalized populations.

Does the article adequately reference differing views and opinions?

Yes

Are all factual statements correct, and are statements and arguments made adequately supported by citations?

Yes

Is the Open Letter written in accessible language?

Yes

Where applicable, are recommendations and next steps explained clearly for others to follow?

Yes

Is the rationale for the Open Letter provided in sufficient detail?

Yes

Reviewer Expertise:

Social Anthropology of nutrition and agricultural development and gender.

I confirm that I have read this submission and believe that I have an appropriate level of expertise to confirm that it is of an acceptable scientific standard.

## References

[1] : Agronomy for Development: The Politics of Knowledge in Agricultural Research. *Routledge Ltd.*.2017; 10.4324/9781315284057 10.4324/9781315284057 Reference source

[2] : Adapting to Climate Uncertainty in African Agriculture.2015; 10.4324/9781315725680 10.4324/9781315725680

# Peer review: Reviewer response for version 1

**DOI:** 10.21956/aasopenres.14174.r27514

**Published:** 2020-07-10

**Authors:** Christopher Huggins

**Affiliations:** 1 School of International Development and Global Studies, University of Ottawa, Ottawa, ON, Canada

This is a timely and well-argued piece on a very important set of issues. Only a few minor revisions are needed, in my view. The following points are offered in the spirit of constructive criticism:

 The article talks of “agricultural technology uptake” and “limited impact at scale”, providing some reasons why this is the case. However, it doesn’t emphasize the overall political economy of corporate and 'donor' involvement in African food systems. To some extent it covers this in the penultimate paragraph: “African agricultural policymaking is biased towards intensifying the production of staple crops, using a narrow range of agrochemicals and improved seeds as evident in multiple Farmer Input Subsidy Programmes”. Nevertheless, perhaps a few sentences on the involvement by agribusiness multinational companies (providing seeds, agrichemical inputs, etc) and philanthropic-public-private partnerships would make for a more critical analysis. Sources such as IPES-Food (2020) *Money Flows: What Is Holding Back Investment In Agroecological Research For Africa?* argues that “As many as 85% of projects funded by the BMGF [Bill and Melinda Gates Foundation] and more than 70% of projects carried out by Kenyan research institutes were limited to supporting industrial agriculture and/or increasing its efficiency via targeted approaches such as improved pesticide practices, livestock vaccines or reductions in post-harvest losses”, rather than more agroecological practices which might address the issues mentioned in this article. A little more critical attention to the political economy of agricultural research – mentioning perhaps the “Green Revolution for Africa” paradigm – would better align the article with its important recommendation that stakeholders “Build the agency of individuals and communities to foster ownership in the management and control of such agricultural innovations”.

 Personally, I would argue for a few sentences at least on the broader agrarian issues involved – based on a brief discussion of (gendered) labour, capital (often invested in patented, profit-making technologies such as biotechnologies) and land. Issues around women’s access to land especially, and land acquisition by large-scale corporations, might deserve attention.

 The following are more minor issues:

 The Abstract talks of the “fragility of our food systems”. While the reader might assume “our” means “African”, it could also be taken to mean “global”. The author should specify.

 The first sentence of the introduction mentions “Various actors” but only includes 1 reference. Another recent reference would be Oxfam’s report ‘The hunger virus: how COVID-19 is fuelling hunger in a hungry world’ https://www.oxfam.org/en/press-releases/12000-people-day-could-die-covid-19-linked-hunger-end-year-potentially-more-disease

 “while current food systems have become efficient at producing and supplying large volumes of food, they have generated multiple adverse outcomes comprising high greenhouse gas emissions, persistent hunger, and livelihood stress for farmers around the world”. To give a broad sense of negative impacts, the author might consider mentioning other environmental impacts (e.g. water pollution, soil degradation, loss of agrobiodiversity).

 “it brings greater scrutiny and critical debate to normative assumptions” – what does the “it” in this sentence refer to? Scholarly and policy debates? The concept of resilience? The increased scrutiny of our food system due to covid-19 impacts?

 “Among them is that most efforts tend to narrowly focus on generating more produce or income from existing farms but pay relatively scarce attention to the different dimensions of agricultural systems constrains” – misspelling of the last word. There is another misspelling (“constrains”) later in the article.

Does the article adequately reference differing views and opinions?

Yes

Are all factual statements correct, and are statements and arguments made adequately supported by citations?

Yes

Is the Open Letter written in accessible language?

Yes

Where applicable, are recommendations and next steps explained clearly for others to follow?

Yes

Is the rationale for the Open Letter provided in sufficient detail?

Yes

Reviewer Expertise:

Food security; political economy of natural resources management in East Africa; land reforms in East and Central Africa

I confirm that I have read this submission and believe that I have an appropriate level of expertise to confirm that it is of an acceptable scientific standard.
